# Bis(3,5-di­nitro­benzoato-κ*O*)bis­(ethane-1,2-di­amine-κ^2^ *N*,*N*′)cadmium(II)

**DOI:** 10.1107/S2414314620008433

**Published:** 2020-07-03

**Authors:** Avazbek Ibragimov

**Affiliations:** a Institute of Bioorganic Chemistry, Academy of Sciences of Uzbekistan, M. Ulugbek Str 83, Tashkent 700125, Uzbekistan; Vienna University of Technology, Austria

**Keywords:** crystal structure, mixed-ligand complex, 3,5-di­nitro­benzoic acid, ethyl­endi­amine

## Abstract

During systematic investigations of bioavailability and biological action enhancement of well known compounds with low bioactivity, a new mixed-ligand metal complex, [Cd(DNBA)_2_(en)_2_)] (DNBA = 3,5-di­nitro­benzoate, C_7_H_3_N_2_O_6_; en = ethyl­endi­amine, C_2_H_8_N_2_), has been synthesized. The complex mol­ecules are located on inversion centers. Two DNBA anions monodentately coordinate the Cd^II^ atom through an oxygen atom of the carboxyl­ate group while two en mol­ecules coordinate in a chelate fashion, resulting in a distorted O_2_N_4_ coordination set. There is a weak intra­molecular hydrogen bond of 3.099 (4) Å between the non-coordinating oxygen atom of the carboxyl­ate group and one of the en amine groups. Three relatively weak inter­molecular N—H⋯O hydrogen bonds associate complex mol­ecules into sheets extending parallel to (0  1), which are further stabilized by *π*–*π* inter­actions. A Hirshfeld surface analysis of the crystal structure indicates that the most important contributions to the crystal packing are from H⋯O/O⋯H (50.2%) and H⋯H (21.1%) inter­actions.

## Abstract 2

In the crystal structure, centrosymmetric mol­ecules are linked through inter­molecular N—H⋯O hydrogen bonds into sheets extending parallel to (0 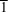 1).

## Graphical abstract

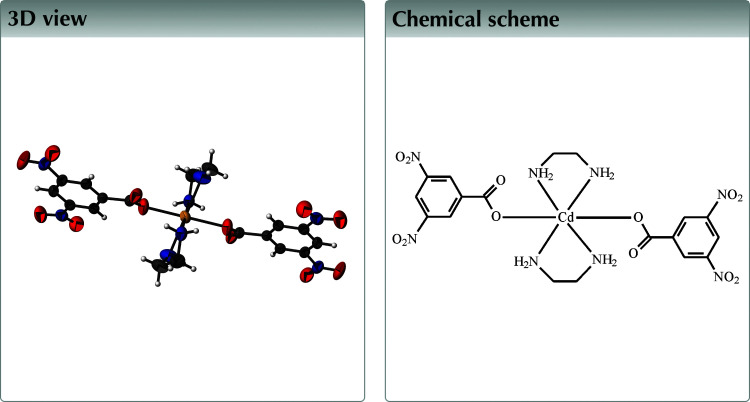

## Structure description

DNBA (= 3,5-di­nitro­benzoic acid) is an organic compound that is an important corrosion inhibitor applied in photography and is used by chemists to identify alcohol components in esters and in the fluoro­metric analysis of creatinine (Chandrasekaran *et al.*, 2013 ▸). DNBA demonstrates low anti­microbial activity against bacteria and yeasts with values of the half maximal inhibitory concentration (IC50) and minimum inhibition concentration (MIC) of more than 3 mmol l^−1^ but shows medium biological action against filamentous fungi *M. gypseum* with IC50 and MIC values of 2.1 and 3 mmol l^−1^ (microbicide effect), respectively (Vaskova *et al.*, 2009 ▸).

En (ethyl­endi­amine) is used in large qu­anti­ties for the production of many industrial chemicals. It is a well known bidentate chelating ligand for coordination complexes (Matsushita & Taira, 1999 ▸). En itself is not biologically active against different strains of microorganisms, but its Co^III^ complex demonstrates a strong anti­fungal action against a broad spectrum of *Candida* species (Turecka *et al.*, 2018 ▸).

The water solubility of DNBA is low (1.35 g l^−1^ at 25°C; Rogers & Stovall, 2000 ▸). In order to enhance its water solubility and anti­microbial activity, we tried to apply some of the presently available approaches (Jain *et al.*, 2015 ▸). However, more encouraging is the combination of organic salts, DNBA and en as well as mixed-ligand complexes comprising respective ligands. Promising results have already been achieved in the case of 4-nitro­benzoic acid (Ibragimov *et al.*, 2017 ▸), 4-amino­benzoic acid (Ibragimov *et al.*, 2016 ▸) and 3-hydroxybenzoic acid (Ibragimov, 2016 ▸). A search of the Cambridge Structural Database (Groom *et al.*, 2016 ▸) has revealed that organic salts on the basis of DNBA have already been obtained [refcodes VUJXIH (Nethaji *et al.*, 1992 ▸) and FONCER (Jones *et al.*, 2005 ▸)] and therefore we made another attempt and synthesized a cadmium-based mixed-ligand complex. The choice of Cd is explained by the fact that compounds based on cadmium are toxic for living organisms including fungi.

**Figure 1:**
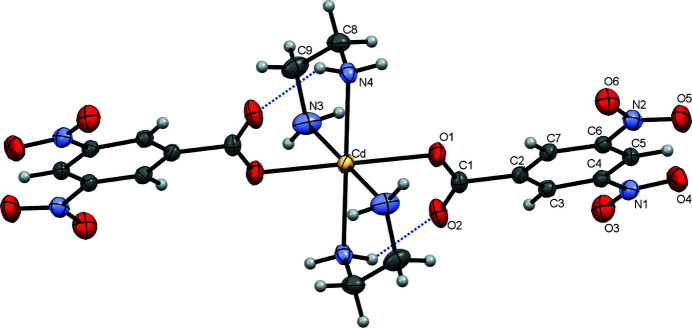
The mol­ecular structure of the coordination complex [Cd(DNBA)_2_(en)_2_)] with displacement ellipsoids shown at the 30% probability level. The crystallographically independent part of the mol­ecule is labelled, the atoms of the remaining part are generated by inversion symmetry. [Symmetry code: (i) −*x* + 2, −*y* + 1, −*z* + 2].

In the crystal of the title compound, the complex mol­ecules are located on inversion centers. Two symmetry-related DNBA anions monodentately coordinate to Cd^II^ through one of the oxygen atoms of the carboxyl­ate group. The two en ligands coordinate in a chelate fashion through the two N atoms (Fig. 1 ▸). The bond lengths Cd—O1, Cd—N3 and Cd—N4 are 2.344 (2), 2.337 (4) and 2.322 (3) Å, respectively, and the *cis*-bond angles vary from 77.34 (12) to 102.66 (12)°, indicating a rather strong distortion from the ideal octa­hedral shape. The conformation of the complex mol­ecule is stabilized through a weak intra­molecular hydrogen bond [3.099 (4) Å and 143 (3)°] between the N4—H4*A* donor and the O2 acceptor (Table 1 ▸) defining a six-membered ring with graph-set notation *S*(6). Most coplanar with the aromatic ring is the N1O_2_ nitro group [dihedral angle of 3.873 (3)°] while the carboxyl­ate group is considerably twisted from the aromatic ring [dihedral angle = 19.332 (9)°]. The arrangement of the N2O_2_ nitro group is inter­mediate between the latter two, the corresponding dihedral angle being 13.529 (6)°.

**Figure 2:**
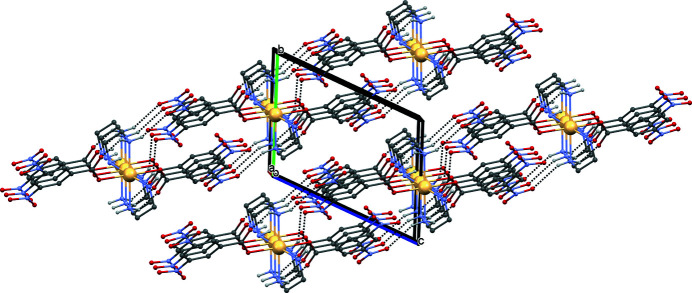
The crystal packing of the coordination complex [Cd(DNBA)_2_(en)_2_)] showing N—H⋯O hydrogen bonds as dashed lines. For clarity, H atoms not involved in hydrogen bonding are omitted.

There are three relatively weak inter­molecular hydrogen bonds in the crystal structure (Table 1 ▸). N4—H4*A*⋯O4^i^ and N4—H4*B*⋯O4^ii^ hydrogen bonds define rings with graph-set notation 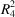 (8). The rings are further connected *via* N3—H3*B*⋯O5^iii^ hydrogen bonds, forming sheets extending parallel to (0 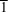 1) (Fig. 2 ▸). The sheets are stabilized by π–π stacking inter­actions [*Cg*1⋯*Cg*1 = 3.715 (3) Å, slippage = 1.608 Å, symmetry operation: 1 − *x*, −*y*, 1 − *z*; *Cg*1 is the centroid of the phenyl (C1–C6) ring].

**Figure 3:**
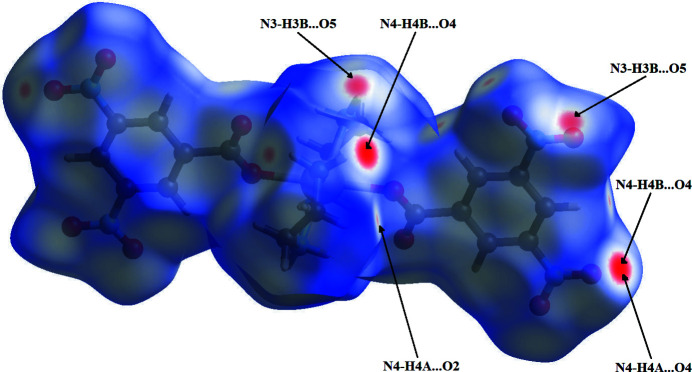
View of the three-dimensional Hirshfeld surface of the title compound plotted over *d*_norm_ in the range −0.2200 to 1.2846 a.u..

**Figure 4:**
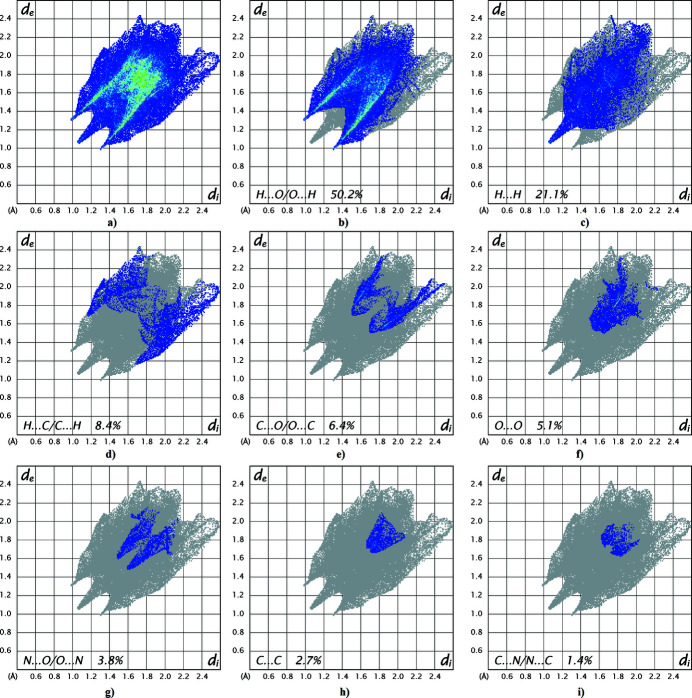
Full two-dimensional fingerprint plots for the title compound, showing (*a*) all inter­actions, and delineated into (*b*) H⋯O/O⋯H, (*c*) H⋯H, (*d*) H⋯C/C⋯H, (*e*) C⋯O/O⋯C, (*f*) O⋯O, (*g*) N⋯O/O⋯N, (*h*) C⋯C and (*i*) C⋯N/N⋯C inter­actions. The *d*_i_ and *d*_e_ values are the closest inter­nal and external distances (in Å) from a given point on the Hirshfeld surface. Relative contributions are indicated.

In order to visualize the inter­molecular inter­actions in the crystal of the title compound, a Hirshfeld surface (HS) analysis was carried out using *Crystal Explorer 17.5* (Turner *et al.*, 2017 ▸). The Hirshfeld surface mapped over *d*_norm_ (Fig. 3 ▸) shows the expected bright-red spots near atoms O2, O4, O5, H3*B*, H4*A* and H4*B* involved in the N—H⋯O hydrogen-bonding inter­actions described above. Fingerprint plots, Fig. 4 ▸, reveal that while H⋯O/O⋯H inter­actions make the greatest contribution to the surface contacts, as would be expected for a mol­ecule with such a predominance of O atoms, H⋯H and H⋯C/C⋯H contacts are also substantial. The C⋯O/O⋯C, O⋯O, N⋯O/O⋯N, C⋯C, C⋯N/N⋯C and H⋯N/N⋯H contacts are less significant.

A search of the Cambridge Structural Database (Version 5.41, November 2019; Groom *et al.*, 2016 ▸) attested that over 300 crystal structures based on DNBA are registered. Among these structures, eleven compounds are monoligand complexes while 120 ones belong to mixed-ligand coordination compounds. There are two mixed-ligand complexes closely related to the [Cd(DNBA)_2_(en)_2_)] complex. The silver complexes with refcodes EQOKEA (Zhu *et al.*, 2003 ▸) and EQOKEA01 (Qiu *et al.*, 2005 ▸) consist of discrete and polymeric components. In the discrete component, Ag^I^ is coordinated by two DNBA mol­ecules in a monodentate mode whereas in the second component silver ions are associated by en ligands into polymeric chains. There are also DNBA, en and –NO_2_ ligands in the Co^I^ complex with refcode KICCEF (Sharma *et al.*, 2007 ▸). In this complex, the metal ion is chelated by two en ligands, and one DNBA and one NO_2_ mol­ecules each in a monodentate mode.

## Synthesis and crystallization

To an aqueous solution (2.5 ml) of Cd(CH_3_COO)_2_ (0.115 g, 0.5 mmol) was slowly added an ethanol solution (4 ml) containing en (60 *μ*l) and DNBA (0.212 g, 1 mmol) under constant stirring. A colourless crystalline product was obtained at room temperature by slow solvent evaporation after 6 d. Single crystals for X-ray structure determination were selected from this product. Yield: 65%. Elemental analysis for C_18_H_22_CdN_8_O_12_ (654.83): calculated C 33.02; H 3.39; N 17.11%; found: C 32.96; H 3.32; N 17.08%.

## Refinement

Crystal data, data collection and structure refinement details are summarized in Table 2 ▸.

## Supplementary Material

Crystal structure: contains datablock(s) I. DOI: 10.1107/S2414314620008433/wm4132sup1.cif

Structure factors: contains datablock(s) I. DOI: 10.1107/S2414314620008433/wm4132Isup2.hkl

CCDC reference: 2011747

Additional supporting information:  crystallographic information; 3D view; checkCIF report

## Figures and Tables

**Table 1:** Hydrogen-bond geometry (Å, °)

*D*—H⋯*A*	*D*—H	H⋯*A*	*D*⋯*A*	*D*—H⋯*A*
N4—H4*A*⋯O2	0.92 (1)	2.32 (2)	3.099 (4)	143 (3)
N4—H4*A*⋯O4^i^	0.92 (1)	2.56 (3)	3.268 (4)	134 (3)
N4—H4*B*⋯O4^ii^	0.92 (1)	2.39 (2)	3.237 (4)	153 (4)
N3—H3*B*⋯O5^iii^	0.92 (1)	2.52 (7)	3.312 (5)	145 (10)

Symmetry codes: (i) 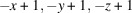 ; (ii) 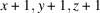 ; (iii) 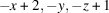 .

**Table 2:** Experimental details

Crystal data	
Chemical formula	[Cd(C_7_H_3_N_2_O_6_)_2_(C_2_H_8_N_2_)_2_)]
*M*_r_	654.83
Crystal system, space group	Triclinic, *P* 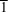
Temperature (K)	291
*a*, *b*, *c* (Å)	7.191 (5), 8.698 (5), 10.987 (5)
α, β, γ (°)	112.289 (5), 92.827 (5), 101.656 (5)
*V* (Å^3^)	616.7 (6)
*Z*	1
Radiation type	Cu *K*α
μ (mm^−1^)	7.81
Crystal size (mm)	0.22 × 0.18 × 0.16

Data collection	
Diffractometer	Rigaku Oxford Diffraction Xcalibur, Ruby
Absorption correction	Multi-scan (*CrysAlis PRO*; Rigaku OD, 2015 ▸)
*T*_min_, *T*_max_	0.397, 1.000
No. of measured, independent and observed [*I* > 2σ(*I*)] reflections	4512, 2482, 2406
*R*_int_	0.031
(sin θ/λ)_max_ (Å^−1^)	0.629

Refinement	
*R*[*F* ^2^ > 2σ(*F* ^2^)], *wR*(*F* ^2^), *S*	0.033, 0.086, 1.06
No. of reflections	2482
No. of parameters	195
No. of restraints	5
H-atom treatment	H atoms treated by a mixture of independent and constrained refinement
Δρ_max_, Δρ_min_ (e Å^−3^)	0.45, −0.51

Computer programs: *CrysAlis PRO* (Rigaku OD, 2015 ▸), *SHELXT* (Sheldrick, 2015*a* ▸), *SHELXL2018/3* (Sheldrick, 2015*b* ▸), *Mercury* (Macrae *et al.*, 2020 ▸) and *publCIF* (Westrip, 2010 ▸).

## full crystallographic data

### Crystal data

**Table:**

[Cd(C_7_H_3_N_2_O_6_)_2_(C_2_H_8_N_2_)_2_)]	*Z* = 1
*M**_r_* = 654.83	*F*(000) = 330
Triclinic, *P*1	*D*_x_ = 1.763 Mg m^−^^3^
*a* = 7.191 (5) Å	Cu *K*α radiation, λ = 1.54184 Å
*b* = 8.698 (5) Å	Cell parameters from 3166 reflections
*c* = 10.987 (5) Å	θ = 4.4–75.1°
α = 112.289 (5)°	µ = 7.81 mm^−^^1^
β = 92.827 (5)°	*T* = 291 K
γ = 101.656 (5)°	Block, colorless
*V* = 616.7 (6) Å^3^	0.22 × 0.18 × 0.16 mm

### Data collection

**Table:**

Rigaku Oxford Diffraction Xcalibur, Ruby diffractometer	2482 independent reflections
Radiation source: fine-focus sealed X-ray tube	2406 reflections with *I* > 2σ(*I*)
Detector resolution: 10.2576 pixels mm^-1^	*R*_int_ = 0.031
ω scans	θ_max_ = 75.8°, θ_min_ = 4.4°
Absorption correction: multi-scan (CrysAlisPro; Rigaku OD, 2015)	*h* = −8→8
*T*_min_ = 0.397, *T*_max_ = 1.000	*k* = −10→10
4512 measured reflections	*l* = −11→13

### Refinement

**Table:**

Refinement on *F*^2^	Secondary atom site location: difference Fourier map
Least-squares matrix: full	Hydrogen site location: mixed
*R*[*F*^2^ > 2σ(*F*^2^)] = 0.033	H atoms treated by a mixture of independent and constrained refinement
*wR*(*F*^2^) = 0.086	*w* = 1/[σ^2^(*F*_o_^2^) + (0.0513*P*)^2^ + 0.2183*P*] where *P* = (*F*_o_^2^ + 2*F*_c_^2^)/3
*S* = 1.06	(Δ/σ)_max_ < 0.001
2482 reflections	Δρ_max_ = 0.45 e Å^−^^3^
195 parameters	Δρ_min_ = −0.51 e Å^−^^3^
5 restraints	Extinction correction: SHELXL2018/3 (Sheldrick 2015b), Fc^*^=kFc[1+0.001xFc^2^λ^3^/sin(2θ)]^-1/4^
Primary atom site location: Intrinsic-phasing	Extinction coefficient: 0.0032 (5)

### Special details

**Table:**

Geometry. All esds (except the esd in the dihedral angle between two l.s. planes) are estimated using the full covariance matrix. The cell esds are taken into account individually in the estimation of esds in distances, angles and torsion angles; correlations between esds in cell parameters are only used when they are defined by crystal symmetry. An approximate (isotropic) treatment of cell esds is used for estimating esds involving l.s. planes.
Refinement. N-bound H atoms were located in a difference Fourier map and were refined with bond-length restraints of 0.92 (1) Å.

### Fractional atomic coordinates and isotropic or equivalent isotropic displacement parameters (Å^2^)

**Table:**

	*x*	*y*	*z*	*U*_iso_*/*U*_eq_	
Cd1	1.000000	0.500000	1.000000	0.04436 (14)	
O2	0.6549 (4)	0.5433 (4)	0.7930 (3)	0.0651 (7)	
O1	0.8216 (4)	0.3493 (3)	0.7881 (2)	0.0620 (7)	
O3	0.1233 (4)	0.3373 (4)	0.4291 (3)	0.0715 (8)	
O4	0.1282 (4)	0.1278 (4)	0.2450 (3)	0.0767 (9)	
O6	0.8565 (4)	−0.0927 (4)	0.3594 (3)	0.0730 (8)	
N1	0.1978 (4)	0.2284 (4)	0.3583 (3)	0.0517 (6)	
N4	1.0222 (4)	0.7651 (3)	0.9904 (3)	0.0487 (6)	
O5	0.6643 (5)	−0.1370 (4)	0.1882 (3)	0.0757 (8)	
N2	0.7258 (4)	−0.0609 (4)	0.3057 (3)	0.0516 (6)	
C2	0.6271 (4)	0.3000 (4)	0.5940 (3)	0.0381 (6)	
C3	0.4603 (4)	0.3208 (4)	0.5398 (3)	0.0396 (6)	
H3	0.402141	0.406126	0.589595	0.047*	
C7	0.7155 (4)	0.1743 (4)	0.5164 (3)	0.0395 (6)	
H7	0.827318	0.158989	0.551588	0.047*	
C6	0.6340 (4)	0.0733 (4)	0.3869 (3)	0.0397 (6)	
C4	0.3822 (4)	0.2132 (4)	0.4114 (3)	0.0401 (6)	
C5	0.4662 (4)	0.0883 (4)	0.3320 (3)	0.0426 (6)	
H5	0.411582	0.017367	0.245051	0.051*	
N3	1.2449 (5)	0.5152 (5)	0.8698 (4)	0.0714 (10)	
C1	0.7084 (4)	0.4101 (4)	0.7390 (3)	0.0443 (7)	
C8	1.2033 (6)	0.8097 (5)	0.9427 (4)	0.0622 (9)	
H8A	1.307671	0.851575	1.015179	0.075*	
H8B	1.200122	0.900523	0.912486	0.075*	
C9	1.2380 (7)	0.6594 (6)	0.8321 (5)	0.0725 (11)	
H9A	1.136935	0.622053	0.758114	0.087*	
H9B	1.358522	0.693491	0.802584	0.087*	
H4A	0.922 (4)	0.747 (5)	0.928 (3)	0.050 (10)*	
H4B	1.024 (6)	0.846 (4)	1.074 (2)	0.072 (13)*	
H3A	1.336 (4)	0.586 (4)	0.940 (3)	0.053 (11)*	
H3B	1.285 (10)	0.419 (8)	0.821 (8)	0.27 (6)*	

### Atomic displacement parameters (Å^2^)

**Table:**

	*U*^11^	*U*^22^	*U*^33^	*U*^12^	*U*^13^	*U*^23^
Cd1	0.0539 (2)	0.0461 (2)	0.03613 (18)	0.01547 (13)	0.00951 (12)	0.01752 (13)
O2	0.0524 (14)	0.0699 (16)	0.0509 (13)	0.0237 (12)	−0.0022 (10)	−0.0035 (12)
O1	0.0744 (17)	0.0650 (15)	0.0418 (12)	0.0244 (13)	−0.0096 (11)	0.0144 (11)
O3	0.0556 (15)	0.086 (2)	0.0744 (18)	0.0318 (14)	−0.0012 (13)	0.0279 (16)
O4	0.0756 (19)	0.0665 (17)	0.0712 (18)	0.0104 (14)	−0.0354 (15)	0.0181 (15)
O6	0.0684 (17)	0.0780 (19)	0.0744 (18)	0.0403 (15)	0.0103 (14)	0.0209 (15)
N1	0.0464 (14)	0.0535 (16)	0.0554 (16)	0.0080 (12)	−0.0075 (12)	0.0257 (13)
N4	0.0558 (16)	0.0437 (14)	0.0428 (14)	0.0192 (12)	0.0014 (11)	0.0099 (11)
O5	0.096 (2)	0.0711 (18)	0.0481 (15)	0.0316 (16)	0.0140 (13)	0.0040 (13)
N2	0.0561 (16)	0.0472 (15)	0.0494 (15)	0.0136 (12)	0.0162 (12)	0.0154 (12)
C2	0.0365 (13)	0.0420 (15)	0.0356 (13)	0.0062 (11)	0.0038 (10)	0.0169 (12)
C3	0.0384 (14)	0.0408 (15)	0.0400 (14)	0.0085 (11)	0.0056 (11)	0.0172 (12)
C7	0.0385 (14)	0.0437 (15)	0.0388 (14)	0.0109 (11)	0.0052 (11)	0.0185 (12)
C6	0.0446 (15)	0.0374 (14)	0.0381 (14)	0.0090 (11)	0.0086 (11)	0.0161 (12)
C4	0.0394 (14)	0.0412 (15)	0.0415 (14)	0.0063 (11)	−0.0018 (11)	0.0208 (12)
C5	0.0483 (16)	0.0400 (15)	0.0350 (13)	0.0043 (12)	0.0011 (11)	0.0139 (12)
N3	0.068 (2)	0.073 (2)	0.098 (3)	0.0355 (19)	0.038 (2)	0.047 (2)
C1	0.0386 (14)	0.0523 (17)	0.0350 (14)	0.0083 (12)	0.0034 (11)	0.0112 (13)
C8	0.064 (2)	0.052 (2)	0.074 (2)	0.0088 (16)	0.0048 (18)	0.0329 (19)
C9	0.074 (3)	0.086 (3)	0.082 (3)	0.029 (2)	0.035 (2)	0.052 (2)

### Geometric parameters (Å, º)

**Table:**

Cd1—N4^i^	2.322 (3)	C2—C7	1.397 (4)
Cd1—N4	2.322 (3)	C2—C1	1.524 (4)
Cd1—N3	2.337 (4)	C3—C4	1.374 (4)
Cd1—N3^i^	2.337 (4)	C3—H3	0.9300
Cd1—O1	2.344 (2)	C7—C6	1.379 (4)
Cd1—O1^i^	2.344 (2)	C7—H7	0.9300
O2—C1	1.235 (4)	C6—C5	1.375 (4)
O1—C1	1.257 (4)	C4—C5	1.380 (4)
O3—N1	1.216 (4)	C5—H5	0.9300
O4—N1	1.226 (4)	N3—C9	1.471 (6)
O6—N2	1.218 (4)	N3—H3A	0.914 (10)
N1—C4	1.473 (4)	N3—H3B	0.916 (10)
N4—C8	1.462 (5)	C8—C9	1.485 (6)
N4—H4A	0.916 (10)	C8—H8A	0.9700
N4—H4B	0.917 (10)	C8—H8B	0.9700
O5—N2	1.216 (4)	C9—H9A	0.9700
N2—C6	1.474 (4)	C9—H9B	0.9700
C2—C3	1.388 (4)		
			
N4^i^—Cd1—N4	179.999 (11)	C6—C7—C2	118.8 (3)
N4^i^—Cd1—N3	102.66 (12)	C6—C7—H7	120.6
N4—Cd1—N3	77.34 (12)	C2—C7—H7	120.6
N4^i^—Cd1—N3^i^	77.34 (12)	C5—C6—C7	122.6 (3)
N4—Cd1—N3^i^	102.66 (12)	C5—C6—N2	118.6 (3)
N3—Cd1—N3^i^	180.0	C7—C6—N2	118.8 (3)
N4^i^—Cd1—O1	86.36 (10)	C3—C4—C5	122.8 (3)
N4—Cd1—O1	93.64 (10)	C3—C4—N1	118.4 (3)
N3—Cd1—O1	80.38 (15)	C5—C4—N1	118.8 (3)
N3^i^—Cd1—O1	99.63 (15)	C6—C5—C4	117.1 (3)
N4^i^—Cd1—O1^i^	93.64 (10)	C6—C5—H5	121.5
N4—Cd1—O1^i^	86.36 (10)	C4—C5—H5	121.5
N3—Cd1—O1^i^	99.62 (15)	C9—N3—Cd1	106.2 (2)
N3^i^—Cd1—O1^i^	80.37 (15)	C9—N3—H3A	90 (3)
O1—Cd1—O1^i^	180.00 (8)	Cd1—N3—H3A	95 (3)
C1—O1—Cd1	122.9 (2)	C9—N3—H3B	126 (8)
O3—N1—O4	124.1 (3)	Cd1—N3—H3B	121 (7)
O3—N1—C4	118.6 (3)	H3A—N3—H3B	110 (2)
O4—N1—C4	117.3 (3)	O2—C1—O1	128.6 (3)
C8—N4—Cd1	107.5 (2)	O2—C1—C2	117.4 (3)
C8—N4—H4A	109 (2)	O1—C1—C2	114.0 (3)
Cd1—N4—H4A	105 (2)	N4—C8—C9	111.1 (3)
C8—N4—H4B	109 (3)	N4—C8—H8A	109.4
Cd1—N4—H4B	110 (3)	C9—C8—H8A	109.4
H4A—N4—H4B	117 (4)	N4—C8—H8B	109.4
O5—N2—O6	123.1 (3)	C9—C8—H8B	109.4
O5—N2—C6	118.1 (3)	H8A—C8—H8B	108.0
O6—N2—C6	118.8 (3)	N3—C9—C8	112.9 (4)
C3—C2—C7	119.7 (3)	N3—C9—H9A	109.0
C3—C2—C1	119.7 (3)	C8—C9—H9A	109.0
C7—C2—C1	120.5 (3)	N3—C9—H9B	109.0
C4—C3—C2	118.9 (3)	C8—C9—H9B	109.0
C4—C3—H3	120.5	H9A—C9—H9B	107.8
C2—C3—H3	120.5		
			
C7—C2—C3—C4	1.8 (4)	O4—N1—C4—C5	0.0 (4)
C1—C2—C3—C4	−175.9 (3)	C7—C6—C5—C4	1.5 (4)
C3—C2—C7—C6	0.0 (4)	N2—C6—C5—C4	178.9 (3)
C1—C2—C7—C6	177.8 (3)	C3—C4—C5—C6	0.5 (4)
C2—C7—C6—C5	−1.8 (4)	N1—C4—C5—C6	−177.3 (3)
C2—C7—C6—N2	−179.2 (3)	Cd1—O1—C1—O2	−8.4 (5)
O5—N2—C6—C5	9.9 (4)	Cd1—O1—C1—C2	172.57 (19)
O6—N2—C6—C5	−168.6 (3)	C3—C2—C1—O2	−19.2 (4)
O5—N2—C6—C7	−172.6 (3)	C7—C2—C1—O2	163.0 (3)
O6—N2—C6—C7	8.9 (4)	C3—C2—C1—O1	159.9 (3)
C2—C3—C4—C5	−2.2 (4)	C7—C2—C1—O1	−17.8 (4)
C2—C3—C4—N1	175.6 (3)	Cd1—N4—C8—C9	42.2 (4)
O3—N1—C4—C3	1.3 (5)	Cd1—N3—C9—C8	41.0 (5)
O4—N1—C4—C3	−177.9 (3)	N4—C8—C9—N3	−59.1 (5)
O3—N1—C4—C5	179.2 (3)		

Symmetry code: (i) −*x*+2, −*y*+1, −*z*+2.

### Hydrogen-bond geometry (Å, º)

**Table:**

*D*—H···*A*	*D*—H	H···*A*	*D*···*A*	*D*—H···*A*
N4—H4*A*···O2	0.92 (1)	2.32 (2)	3.099 (4)	143 (3)
N4—H4*A*···O4^ii^	0.92 (1)	2.56 (3)	3.268 (4)	134 (3)
N4—H4*B*···O4^iii^	0.92 (1)	2.39 (2)	3.237 (4)	153 (4)
N3—H3*B*···O5^iv^	0.92 (1)	2.52 (7)	3.312 (5)	145 (10)

Symmetry codes: (ii) −*x*+1, −*y*+1, −*z*+1; (iii) *x*+1, *y*+1, *z*+1; (iv) −*x*+2, −*y*, −*z*+1.

### Percentage contributions to the Hirshfeld surface for (I).

**Table:**

Contacts	Included surface area %
H···O/O···H	50.2
H···H	21.1
H···C/C···H	8.4
C···O/O···C	6.4
O···O	5.1
N···O/O···N	3.8
C···C	2.7
C···N/N···C	1.4
H···N/N···H	1.0
